# Construction of a nomogram combining CT and serum markers for predicting macrolide resistance gene mutation status in pediatric Mycoplasma pneumoniae pneumonia

**DOI:** 10.3389/fped.2026.1828908

**Published:** 2026-05-08

**Authors:** Xinhui Yuan, Rui Xin, Lifang Wang, Jizu Ling, Yumei Li, Lihong Pang

**Affiliations:** Department of Pediatrics, the First Hospital of Lanzhou University, The First Clinical Medical College of Lanzhou University, Lanzhou, Gansu Province, China

**Keywords:** biomarkers, computed tomography, macrolide resistance, Mycoplasma pneumoniae, nomogram

## Abstract

**Purpose:**

To develop and validate a nomogram integrating chest computed tomography (CT) features and serum biomarkers for predicting the mutation status of macrolide resistance genes in pediatric Mycoplasma pneumoniae pneumonia (MPP).

**Methods:**

This retrospective study enrolled children diagnosed with MPP, genetically stratified into macrolide-resistant (MRMP) and macrolide-sensitive (MSMP) groups based on 23S rRNA gene mutations (A2063G/A2064G). Clinical data, chest CT scans obtained within 48 h of admission, and serum biomarker levels (including C-reactive protein [CRP], procalcitonin [PCT], interleukin-6 [IL-6], D-dimer, neutrophil-to-lymphocyte ratio [NLR], and lactate dehydrogenase to albumin ratio [LAR]) measured within 24 h of admission prior to antibiotic therapy were analyzed.

**Results:**

Compared to the MSMP group (*n* = 283), the MRMP group (*n* = 145) had longer fever duration (5.91 vs. 4.85 days) and hospital stay (8.77 vs. 7.32 days), higher rates of shortness of breath (35.86% vs. 24.38%) and oxygen requirement (25.52% vs. 13.78%), elevated serum levels of CRP (39.16 vs. 31.74 mg/L), D-dimer (1.51 vs. 1.32 mg/L FEU), NLR (6.15 vs. 5.22), PCT (0.55 vs. 0.35 ng/mL), IL-6 (28.86 vs. 21.35 pg/mL), and LAR (9.19 vs. 8.42), and more frequent CT findings of lobar consolidation (68.28% vs. 45.23%), bilateral involvement (66.21% vs. 51.59%), pleural effusion (32.41% vs. 14.49%), and higher total severity score (9.22 vs. 7.88). The nomogram based on these independent predictors showed excellent discrimination with an area under the curve of 0.869.

**Conclusion:**

A nomogram combining CT imaging characteristics and serum biomarkers predicted mutation status of macrolide resistance genes in pediatric MPP, potentially aiding early clinical decision-making.

## 1 Introduction

Mycoplasma pneumoniae (M. pneumoniae) is a leading cause of community-acquired pneumonia in children. Traditionally, macrolides have been the first-line treatment due to their efficacy and safety profile (1, 2). However, the emergence of macrolide-resistant strains poses a significant therapeutic challenge, necessitating more accurate methods for early identification and management. Resistance primarily arises from point mutations in the 23S rRNA gene, which impair the binding affinity of macrolides to bacterial ribosomes, thereby reducing their effectiveness. The prevalence of macrolide resistance has been increasing globally, particularly in China (3, 4). This underscores the urgent need for improved diagnostic tools that can predict macrolide resistance before initiating treatment.

Clinical features alone are often insufficient for distinguishing between macrolide-sensitive and macrolide-resistant M. pneumoniae infections. Symptoms such as fever, cough, and respiratory distress can be similar across both groups, complicating clinical decision-making (5–7). Laboratory tests, including white blood cell count and C-reactive protein (CRP), offer some insights but lack the specificity needed to reliably predict macrolide resistance. Consequently, there is a growing interest in leveraging advanced biomarkers and imaging techniques to enhance predict accuracy. Currently, genetic testing for 23S rRNA gene mutations (e.g., PCR-based melt curve analysis) is the gold standard for confirming macrolide resistance. However, this method is time-consuming (typically 24–48 h), requires specialized equipment and trained personnel, and is not universally available in resource-limited settings. These limitations hinder its utility for early, point-of-care clinical decision-making. Procalcitonin (PCT) and interleukin-6 (IL-6) have emerged as promising indicators of severe bacterial infections and systemic inflammation, respectively. These markers could potentially differentiate between resistant and sensitive strains by reflecting the extent of immune activation and disease severity (8–10). Additionally, CT imaging provides detailed anatomical information about lung involvement, which may correlate with the presence of macrolide resistance.

CT scans reveal various patterns of lung involvement in M. pneumoniae pneumonia, including ground-glass opacities, consolidation, and pleural effusion. These radiological findings not only guide diagnosis but also help assess disease severity and progression. For instance, extensive consolidation and bilateral lung involvement are associated with more severe disease and poorer outcomes (11–13). Given the potential link between these radiological features and macrolide resistance, integrating CT imaging with serum biomarkers could offer a more comprehensive risk assessment tool. This dual approach addresses the limitations of relying on either method alone, providing a holistic view of the patient's condition (14, 15).

The rising prevalence of macrolide-resistant M. pneumoniae pneumonia, driven by 23S rRNA gene mutations, necessitates innovative approaches for early detection and management. The combination of CT imaging and serum biomarkers holds potential for improving the prediction of macrolide resistance gene mutation status in pediatric patients, thereby enabling timely alternative antibiotic therapy without awaiting genetic testing results. By developing a robust nomogram that integrates these variables, clinicians may achieve more accurate risk stratification, enabling personalized treatment strategies. This approach addresses the limitations of current diagnostic methods and paves the way for optimized therapeutic interventions. Further research is warranted to validate these findings and explore the broader implications of this integrated model in clinical settings.

## 2 Materials and methods

### 2.1 Study design and participants

This retrospective study was conducted at the First Hospital of Lanzhou University and reviewed the medical records of 428 pediatric patients diagnosed with Mycoplasma pneumoniae pneumonia (M. pneumoniae Pneumonia) between January 2018 and October 2025. The diagnosis of M. pneumoniae Pneumonia required meeting all of the following criteria (16): (1) clinical symptoms and signs consistent with community-acquired pneumonia (e.g., fever, cough, tachypnea, or abnormal auscultatory findings); (2) radiological evidence of pneumonia on chest x-ray or computed tomography (CT); and (3) microbiological confirmation, defined as a positive polymerase chain reaction (PCR) result for M. pneumoniae DNA from a nasopharyngeal swab or bronchoalveolar lavage fluid (BALF).

Patients were excluded if they met any of the following criteria: (1) age > 18 years; (2) presence of immunodeficiency, chronic cardiopulmonary, hepatic, or renal diseases; (3) concurrent active infection with other primary bacterial, viral, or fungal pathogens at admission; (4) incomplete medical records or absence of a chest CT scan within 48 h of admission; (5) having received systemic corticosteroids or other immunomodulators prior to admission.

### 2.2 Data collection

Demographic data, detailed clinical history, physical examination findings on admission, laboratory results, and imaging findings were extracted from electronic medical records using a standardized data collection form. The study was approved by the Institutional Review Board of the First Hospital of Lanzhou University (Approval No.LDYYLL2026-43), and the requirement for informed consent was waived due to its retrospective nature.

### 2.3 Definition of macrolide resistance and grouping

Macrolide resistance was genetically defined by detecting point mutations in domain V of the 23S ribosomal RNA (rRNA) gene of M. pneumoniae. DNA extracted from respiratory specimens (nasopharyngeal swabs or BALF) was analyzed using a commercial real-time PCR-based melt curve analysis kit (Sansure Biotech, China) specifically designed to identify the A2063G and A2064G mutations, which account for over 90% of macrolide resistance in China. The testing was performed according to the manufacturer’s instructions on a real-time PCR system (Bio-Rad CFX96). Patients were defined into two groups based on macrolide resistance gene mutation status: the Macrolide-Resistant M. pneumoniae Pneumonia (MRMP) group and the Macrolide-Sensitive M. pneumoniae Pneumonia (MSMP) group. Patients harboring M. pneumoniae with either the A2063G or A2064G mutation were classified into the MRMP group. Patients with M. pneumoniae lacking these mutations were classified into the MSMP group. The primary endpoint of this study was the presence of A2063G or A2064G mutations, not clinical treatment response.

### 2.4 CT image acquisition and evaluation

Non-contrast chest CT scans were performed on all enrolled patients within 48 h of admission using standardized protocols on 64-slice or higher multi-detector CT scanners (Siemens SOMATOM Perspective, GE Revolution ACT, or Philips Ingenuity Core). Scanning parameters were as follows: tube voltage 100–120 kVp, automatic tube current modulation, slice thickness 1.0 mm, and reconstruction with both lung and mediastinal windows. All CT scans were performed using age-appropriate, weight-based protocols in accordance with the “as low as reasonably achievable” (ALARA) principle. Tube voltage was set at 100 kVp for children under 6 years of age and 120 kVp for older children, with automatic tube current modulation applied in all cases to minimize radiation exposure while maintaining diagnostic image quality. All CT images were independently reviewed by board-certified pediatric radiologists with over 5 years of experience, who were blinded to the patients’ clinical data, laboratory results, and resistance status. The evaluation comprised both qualitative and quantitative assessments. Qualitatively, the readers recorded the presence or absence of the following imaging features: lobar or lobular consolidation, ground-glass opacities, bronchial wall thickening, bilateral lung involvement, and pleural effusion. Quantitatively, a modified CT Total Severity Score (TSS) was applied. Each of the five lung lobes was visually scored from 0 to 4 based on the approximate proportion of parenchymal involvement (0: 0%; 1: 1%–25%; 2: 26%–50%; 3: 51%–75%; 4: 76%–100%). The scores from all five lobes were summed to yield a total score ranging from 0 to 20, with higher scores indicating more extensive disease (17). To assess interobserver reliability, the intraclass correlation coefficient (ICC) was calculatedusing a two-way random-effects model for absolute agreement. The ICC for the TSS was 0.92 (95% CI: 0.88–0.95), indicating excellent consistency between the two radiologists. For qualitative features (e.g., consolidation, pleural effusion), interobserver agreement was assessed using Cohen's kappa coefficient, which ranged from 0.85 to 0.93 across different features, indicating substantial to excellent agreement. Discrepancies in qualitative findings or TSS differences greater than 2 points were resolved through a joint re-evaluation session to reach a final consensus.

### 2.5 Measurement of Serum biomarkers

Fasting venous blood samples were collected from all patients within 24 h of admission and prior to the initiation of any antibiotic therapy. Serum was separated by centrifugation and analyzed immediately or stored at −80 °C for batch testing. All laboratory measurements were performed in the hospital’s central clinical laboratory following standardized operating procedures. The white blood cell count (WBC), differential neutrophil and lymphocyte percentages, and platelet count were determined using an automated hematology analyzer (Sysmex XN-9000, Japan). The neutrophil-to-lymphocyte ratio (NLR) was calculated by dividing the absolute neutrophil count by the absolute lymphocyte count. Serum C-reactive protein (CRP) levels were measured by immunoturbidimetry (Mindray, China, model BS-2000M). Procalcitonin (PCT) and Interleukin-6 (IL-6) levels were quantified using electrochemiluminescence immunoassays on a fully automated analyzer (Roche Cobas e801, Switzerland). Albumin and lactate dehydrogenase (LDH) were assessed using enzymatic methods on a fully automated biochemical analyzer (Beckman Coulter AU5800, USA). D-dimer levels were measured by an immunoturbidimetric assay (Siemens Atellica COAG 360 analyzer, Germany). The lactate dehydrogenase to albumin ratio (LAR) was subsequently calculated by dividing the LDH value (U/L) by the albumin value (g/L).

### 2.6 Statistical analysis

The sample size was determined *a priori* using G*Power software (version 3.1.9.7). Assuming a medium effect size (Cohen's d = 0.5), a two-tailed significance level (*α* = 0.05), and a power of 0.95 for an independent samples t-test, the minimum required sample size was calculated to be 105 patients per group. The final enrollment of 283 patients in the macrolide-sensitive group and 145 in the macrolide-resistant group reflects the natural epidemiological distribution of macrolide resistance in our region. This imbalance, while methodologically acknowledged, does not compromise the statistical power for detecting significant differences in the primary outcomes.

Statistical analyses were performed using SPSS (version 29.0, IBM Corp.) and R software (version 4.0.0). Continuous variables were tested for normality using the Shapiro–Wilk test. Normally distributed data were presented as mea*n* ± standard deviation and compared using the independent samples t-test. Categorical variables were presented as counts (percentages) and compared using the Chi-square test. Variables with a *P* value < 0.05 in the univariate analysis were entered into a least absolute shrinkage and selection operator (LASSO) regression model to further evaluate variable selection and avoid overfitting. The optimal regularization parameter *λ* was determined via 10-fold cross-validation based on the minimum mean squared error. Although all 14 variables retained non-zero coefficients at the optimal *λ*, the LASSO procedure served to regularize the coefficients and mitigate overfitting, confirming the robustness of the subsequent multivariable model. Subsequently, multivariable binary logistic regression analysis (forward stepwise method) was performed to identify independent predictors. The results were expressed as odds ratios (OR) with 95% confidence intervals (CI). A nomogram was constructed based on the final multivariate model using the rms package in R. For the final nomogram, we performed 10-fold cross-validation to assess model stability and generalizability. The discriminative ability of the nomogram was evaluated by the cross-validated area under the receiver operating characteristic curve (AUC) and its 95% CI. Calibration was assessed using a calibration plot with 1000 bootstrap resamples, and decision curve analysis was performed to evaluate the clinical net benefit of the nomogram across a range of threshold probabilities. Multicollinearity among the independent predictors was assessed using the variance inflation factor (VIF), with a VIF < 5 indicating no significant collinearity. All tests were two-tailed, and a *P* value < 0.05 was considered statistically significant.

## 3 Results

### 3.1 Study population and baseline characteristics

The demographic and baseline characteristics of the MSMP and MRMP groups are summarized in Table 1. No significant differences were observed between the two groups regarding age, sex distribution, BMI, season of onset, history of atopy, or prior macrolide use within 3 months (all *P* > 0.05). However, the duration of fever prior to admission and the total hospital stay were significantly longer in the MRMP group compared to the MSMP group (5.91 ± 1.78 vs. 4.85 ± 1.42 days, *P* < 0.001; and 8.77 ± 2.55 vs. 7.32 ± 2.28 days, *P* < 0.001, respectively).

**Table 1:** Demographic and baseline clinical characteristics.

Parameters	MSMP Group (*n* = 283)	MRMP Group (*n* = 145)	t/*χ*^2^	P
Age (years)	7.23 ± 2.48	7.48 ± 2.61	0.974	0.330
Sex (Male), *n* (%)	161 (56.89%)/122 (43.11%)	78 (53.79%)/67 (46.21%)	0.373	0.541
BMI (kg/m^2^)	16.79 ± 2.54	16.55 ± 2.43	0.955	0.340
Season of Onset, *n* (%)			0.269	0.966
- Spring	81 (28.62%)	39 (26.90%)		
- Summer	59 (20.85%)	33 (22.76%)		
- Autumn	77 (27.21%)	39 (26.90%)		
- Winter	66 (23.32%)	34 (23.45%)		
History of Atopy, *n* (%)	63 (22.26%)/220 (77.74%)	39 (26.9%)/106 (73.1%)	1.135	0.287
Prior Macrolide Use (<3 months), *n* (%)	53 (18.73%)/230 (81.27%)	35 (24.14%)/110 (75.86%)	1.718	0.190
Fever Duration Pre-Admission (days)	4.85 ± 1.42	5.91 ± 1.78	6.184	<0.001
Hospital Stay (days)	7.32 ± 2.28	8.77 ± 2.55	5.965	<0.001

BMI, body Mass Index; MSMP, Macrolide-Sensitive Mycoplasma pneumoniae pneumonia; MRMP, Macrolide-Resistant Mycoplasma pneumoniae pneumonia.

Clinical symptoms and signs on admission are compared in Table 2. While the prevalence of cough and extrapulmonary complications did not differ between groups (*P* > 0.05), a significantly higher proportion of patients in the MRMP group presented with shortness of breath (35.86% vs. 24.38%, *P* = 0.013) and required oxygen therapy at admission (25.52% vs. 13.78%, *P* = 0.003).

**Table 2:** Comparison of clinical symptoms and signs on admission.

Parameters	MSMP Group (*n* = 283)	MRMP Group (*n* = 145)	χ^2^	P
Cough, *n* (%)	278 (98.23%)	144 (99.31%)	0.214	0.644
Shortness of Breath, *n* (%)	69 (24.38%)	52 (35.86%)	6.231	0.013
Oxygen Therapy Required, *n* (%)	39 (13.78%)	37 (25.52%)	9.043	0.003
Extrapulmonary Complications^a^, *n* (%)	36 (12.72%)	23 (15.86%)	0.796	0.372

MSMP, Macrolide-Sensitive Mycoplasma pneumoniae pneumonia; MRMP, Macrolide-Resistant Mycoplasma pneumoniae pneumonia.

a Extrapulmonary complications included rash, myocarditis, hepatitis, hemolytic anemia, and neurological symptoms.

### 3.2 Laboratory findings

As shown in Table 3, routine laboratory parameters including WBC, neutrophil percentage, lymphocyte percentage, and platelet count were comparable between the MSMP and MRMP groups (all *P* > 0.05). In contrast, admission levels of CRP, D-dimer, and the calculated NLR were significantly elevated in the MRMP group (39.16 ± 16.88 vs. 31.74 ± 12.23 mg/L, *P* < 0.001; 1.51 ± 0.71 vs. 1.32 ± 0.62 mg/L FEU, *P* = 0.006; and 6.15 ± 2.68 vs. 5.22 ± 2.35, *P* < 0.001, respectively).

**Table 3:** Comparison of routine laboratory indicators on admission.

Parameters	MSMP Group (*n* = 283)	MRMP Group (*n* = 145)	t	P
WBC (×10^9^/L)	9.22 ± 2.61	9.65 ± 2.35	1.678	0.094
Neutrophils (%)	65.83 ± 13.15	67.54 ± 12.83	1.278	0.202
Lymphocytes (%)	26.22 ± 11.35	24.81 ± 10.56	1.243	0.214
Platelets (×10^9^/L)	281.54 ± 95.86	295.37 ± 106.78	1.358	0.175
CRP (mg/L)	31.74 ± 12.23	39.16 ± 16.88	4.697	<0.001
D-dimer (mg/L FEU)	1.32 ± 0.62	1.51 ± 0.71	2.778	0.006
NLR	5.22 ± 2.35	6.15 ± 2.68	3.688	<0.001

WBC, White blood cell count; CRP, C-reactive protein; LDH, lactate dehydrogenase; NLR, neutrophil-to-lymphocyte ratio; MSMP, Macrolide-Sensitive Mycoplasma pneumoniae pneumonia; MRMP, Macrolide-Resistant Mycoplasma pneumoniae pneumonia.

### 3.3 Selected serum biomarkers associated with macrolide resistance gene mutation status

**Figure 1:**
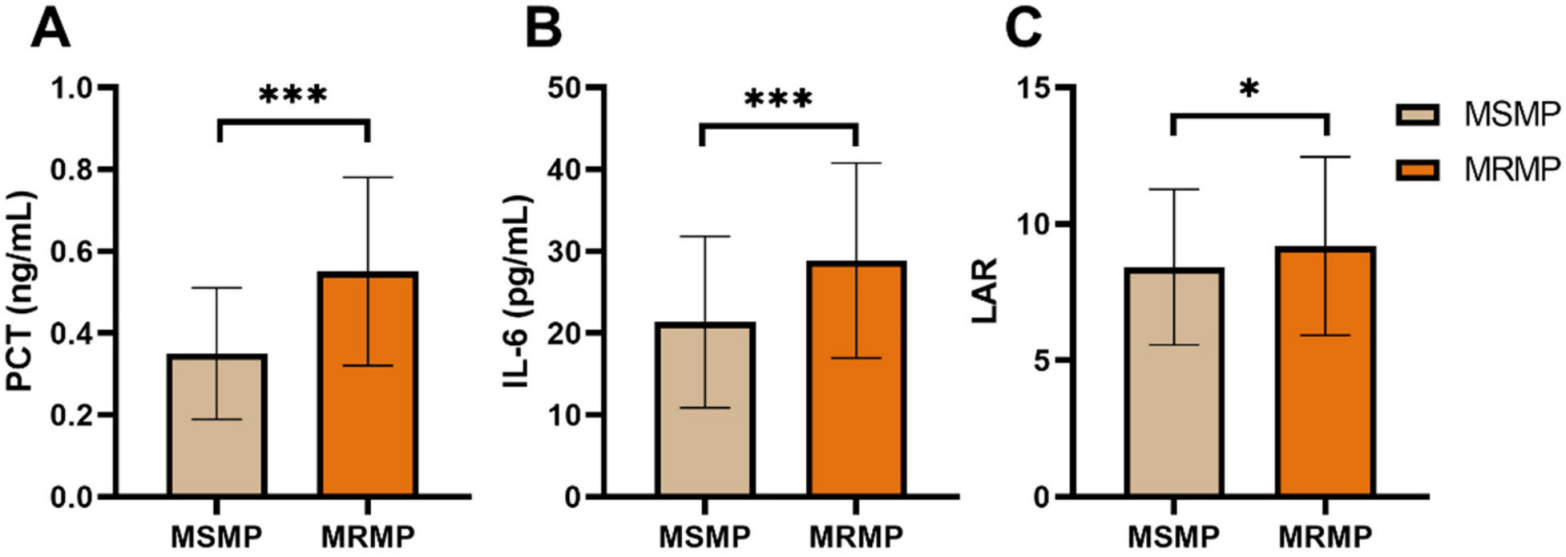
Comparison of selected Serum biomarkers according to macrolide resistance gene mutation Status. **(A)** PCT; **(B)** IL-6; **(C)** LAR.

The comparison of selected serum biomarkers according to macrolide resistance gene mutation status is presented in Figure 1. Serum levels of PCT and IL-6, as well as the LAR, were all significantly higher in the MRMP group than in the MSMP group (0.55 ± 0.23 vs. 0.35 ± 0.16 ng/mL, *P* < 0.001; 28.86 ± 11.91 vs. 21.35 ± 10.47 pg/mL, *P* < 0.001; and 9.19 ± 3.27 vs. 8.42 ± 2.85, *P* = 0.013, respectively).

### 3.4 CT imaging characteristics

**Figure 2:**
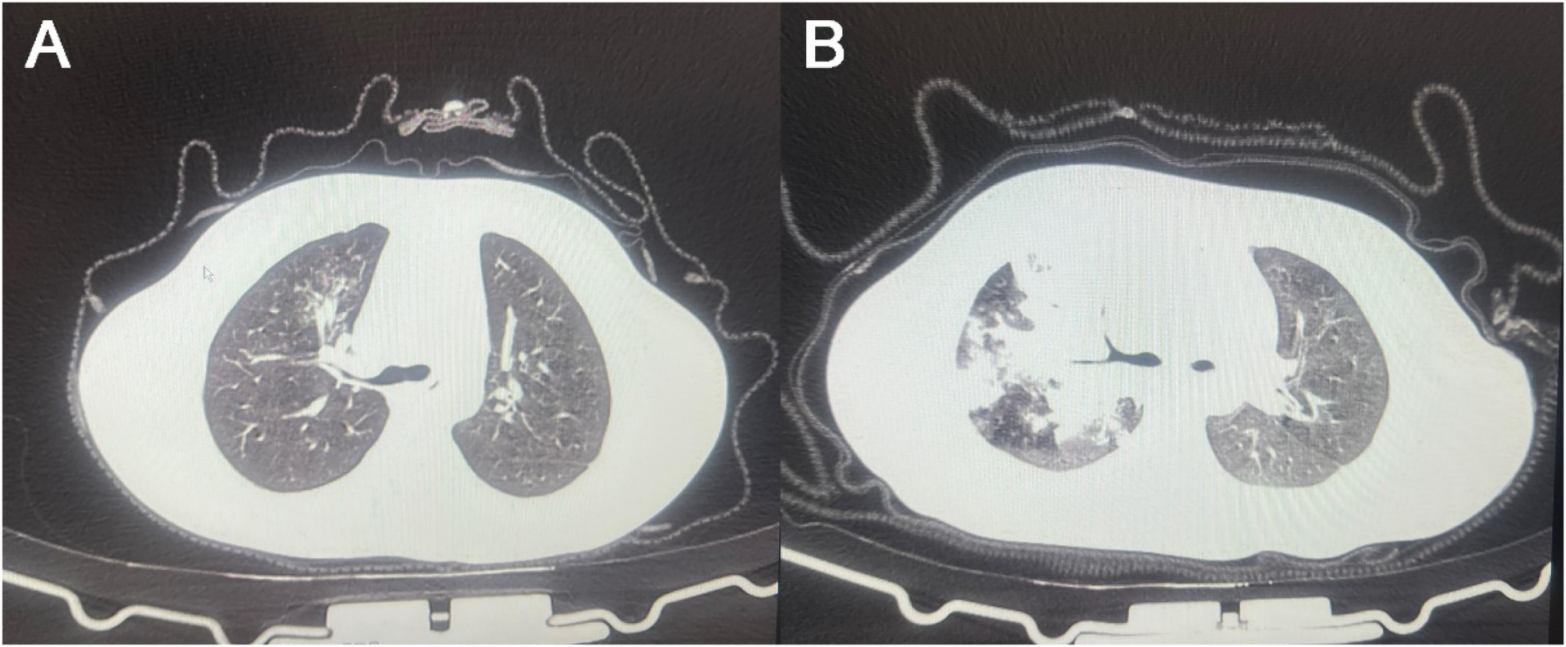
Representative chest CT images from the **(A)** MSMP group and **(B)** MRMP group. PCT, Procalcitonin; IL-6, Interleukin-6; LAR, lactate dehydrogenase to albumin ratio; MSMP, Macrolide-Sensitive Mycoplasma pneumoniae pneumonia; MRMP, Macrolide-Resistant Mycoplasma pneumoniae pneumonia. *: *P* < 0.05; ***: *P* < 0.001.

CT imaging features are detailed in Table 4 and Figure 2. The presence of ground-glass opacities and bronchial wall thickening was similar between the two groups (*P* > 0.05). Notably, lobar/lobular consolidation, bilateral lung involvement, pleural effusion, and the CT TSS were all more frequent or severe in the MRMP group (68.28% vs. 45.23%, *P* < 0.001; 66.21% vs. 51.59%, *P* = 0.004; 32.41% vs. 14.49%, *P* < 0.001; and 9.22 ± 3.41 vs. 7.88 ± 2.95, *P* < 0.001, respectively).

**Table 4:** Comparison of CT imaging features.

Parameters	MSMP Group (*n* = 283)	MRMP Group (*n* = 145)	t/χ^2^	P
Lobar/Lobular Consolidation, *n* (%)	128 (45.23%)	99 (68.28%)	20.444	<0.001
Ground-Glass Opacities, *n* (%)	215 (75.97%)	110 (75.86%)	0.001	0.980
Bronchial Wall Thickening, *n* (%)	258 (91.17%)	135 (93.1%)	0.479	0.489
Bilateral Involvement, *n* (%)	146 (51.59%)	96 (66.21%)	8.336	0.004
Pleural Effusion, *n* (%)	41 (14.49%)	47 (32.41%)	18.863	<0.001
CT Total Severity Score (TSS)	7.88 ± 2.95	9.22 ± 3.41	4.022	<0.001

CT, computed tomography; TSS, Total Severity Score; MSMP, Macrolide-Sensitive Mycoplasma pneumoniae pneumonia; MRMP, Macrolide-Resistant Mycoplasma pneumoniae pneumonia.

### 3.5 Univariate and multivariate analysis of factors associated with macrolide resistance

Univariate logistic regression analysis identified multiple factors associated with macrolide resistance, as detailed in Table 5. Significant variables included longer fever duration and hospital stay, clinical signs (shortness of breath, need for oxygen therapy), elevated serum markers (CRP, D-dimer, NLR, PCT, IL-6, LAR), and specific CT findings (lobar/lobular consolidation, bilateral involvement, pleural effusion, higher TSS) (all *P* < 0.05).

**Table 5:** Univariate analysis of factors associated with macrolide resistance.

Parameters	Coefficient	Std_Error	Wald	P_Value	OR	CI_Lower	CI_Upper
Fever Duration Pre-Admission (days)	0.443	0.073	6.038	<0.001	1.557	1.354	1.805
Hospital Stay (days)	0.260	0.047	5.525	<0.001	1.297	1.185	1.426
Shortness of Breath, *n* (%)	0.551	0.222	2.483	0.013	1.734	1.121	2.677
Oxygen Therapy Required, *n* (%)	0.762	0.257	2.967	0.003	2.143	1.293	3.551
CRP (mg/L)	0.037	0.008	4.891	<0.001	1.038	1.023	1.054
D-dimer (mg/L FEU)	0.432	0.158	2.736	0.006	1.540	1.133	2.106
NLR	0.152	0.042	3.581	<0.001	1.164	1.072	1.267
PCT (ng/mL)	5.689	0.684	8.319	<0.001	9.699	5.695	18.536
IL-6 (pg/mL)	0.061	0.010	6.083	<0.001	1.063	1.043	1.085
LAR	0.085	0.034	2.477	0.013	1.089	1.019	1.166
Lobar/Lobular Consolidation, *n* (%)	0.958	0.215	4.461	<0.001	2.606	1.719	3.994
Bilateral Involvement, *n* (%)	0.609	0.212	2.871	0.004	1.838	1.218	2.800
Pleural Effusion, *n* (%)	1.041	0.245	4.248	<0.001	2.831	1.754	4.591
CT Total Severity Score (TSS)	0.137	0.034	4.058	<0.001	1.147	1.074	1.227

**Figure 3:**
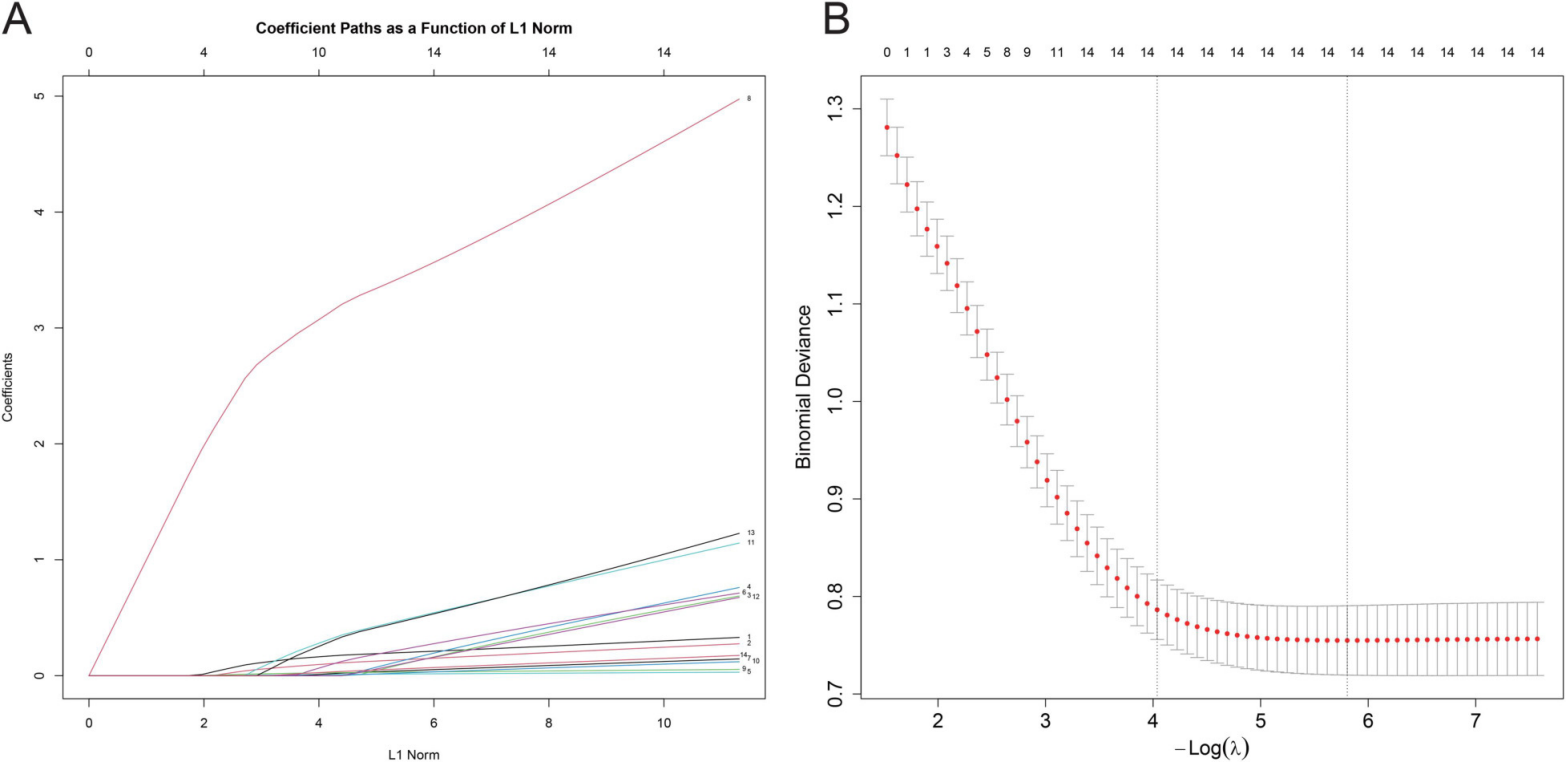
Feature selection via LASSO regression for predicting macrolide resistance. **(A)** Coefficient profiles of the 14 variables across the L1 norm spectrum. Each colored line represents a variable's coefficient trajectory. **(B)** Cross-validation for optimal *λ* selection. The left vertical dashed line indicates the *λ* value that minimizes the mean squared error (*λ*_min), at which all 14 variables retained non-zero coefficients.

To address potential overfitting, LASSO regression was applied to the 14 variables that were significant in univariate analysis. The coefficient profiles of these variables across the L1 norm are shown in Figure 3A. Cross-validation based on the minimum mean squared error selected the optimal *λ* value (*λ*_min), at which all 14 variables retained non-zero coefficients (Figure 3B), indicating that each variable contributed meaningfully to the prediction of macrolide resistance. These results support the robustness of the subsequent multivariable logistic regression model.

Multivariate logistic regression analysis confirmed these factors as independent risk factors of macrolide resistance (Table 6). The final model retained fever duration, hospital stay, shortness of breath, need for oxygen therapy, CRP, D-dimer, NLR, PCT, IL-6, LAR, lobar/lobular consolidation, bilateral involvement, pleural effusion, and CT TSS (all *P* < 0.05).

**Table 6:** Multivariate logistic regression analysis of independent risk factors.

Parameters	Coefficient	Std_Error	Wald_Stat	P_Value	OR	OR_CI_Lower	OR_CI_Upper
Fever Duration Pre-Admission (days)	0.334	0.097	3.439	<0.001	1.397	1.155	1.690
Hospital Stay (days)	0.279	0.065	4.317	<0.001	1.322	1.164	1.500
Shortness of Breath, *n* (%)	0.703	0.321	2.189	0.029	2.019	1.076	3.789
Oxygen Therapy Required, *n* (%)	0.779	0.386	2.021	0.043	2.179	1.024	4.640
CRP (mg/L)	0.030	0.011	2.819	0.005	1.031	1.009	1.052
D-dimer (mg/L FEU)	0.726	0.223	3.250	0.001	2.068	1.334	3.204
NLR	0.147	0.060	2.463	0.014	1.158	1.031	1.302
PCT (ng/mL)	5.025	0.845	5.951	<0.001	9.243	4.084	19.923
IL-6 (pg/mL)	0.055	0.014	3.968	<0.001	1.056	1.028	1.085
LAR	0.123	0.050	2.464	0.014	1.131	1.025	1.247
Lobar/Lobular Consolidation, *n* (%)	1.164	0.315	3.696	<0.001	3.201	1.727	5.934
Bilateral Involvement, *n* (%)	0.692	0.309	2.240	0.025	1.998	1.090	3.663
Pleural Effusion, *n* (%)	1.253	0.385	3.255	0.001	3.500	1.646	7.443
CT Total Severity Score (TSS)	0.178	0.050	3.547	<0.001	1.195	1.083	1.318

### 3.6 Construction of the nomogram

**Figure 4:**
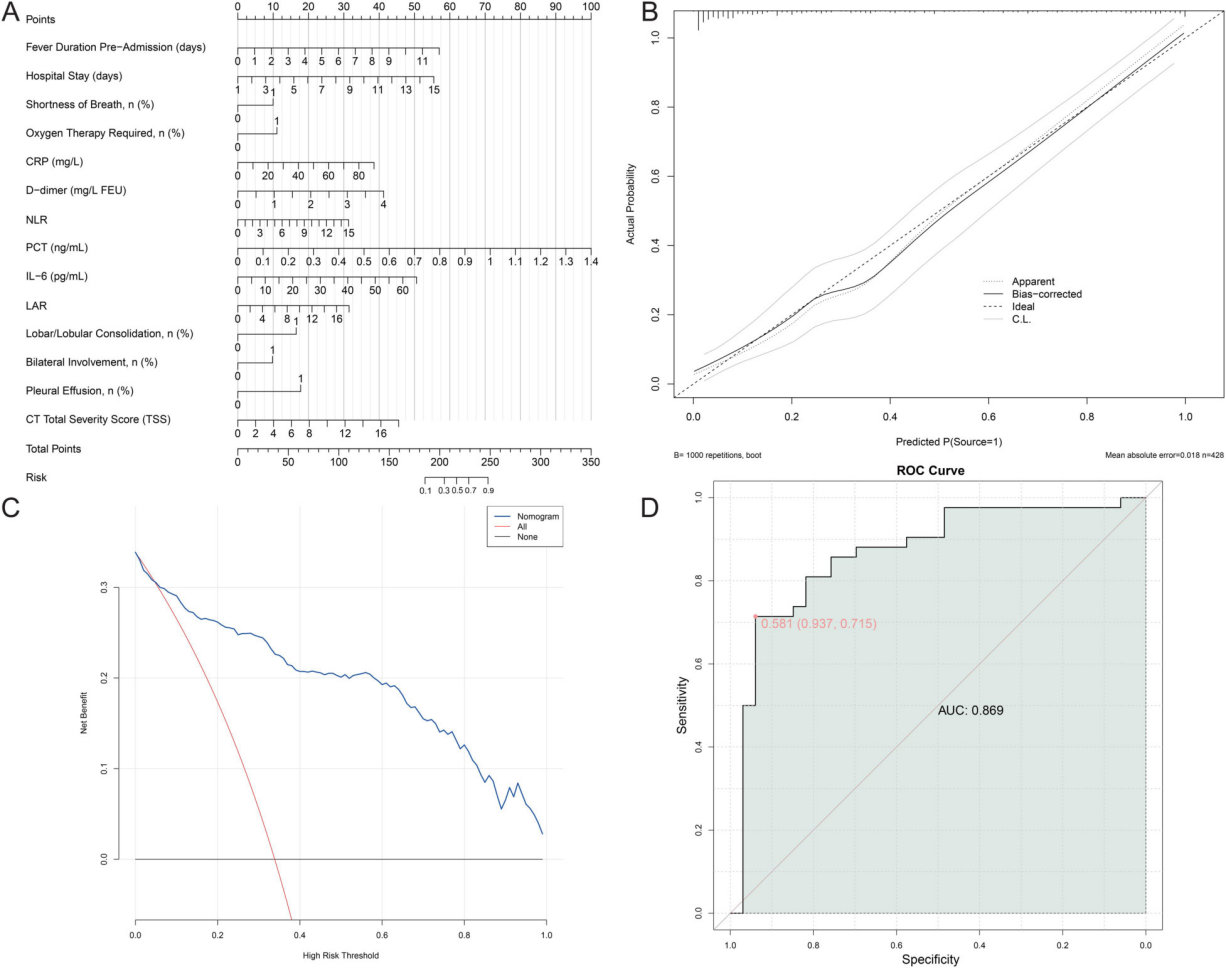
Predictive model for predicting the risk of macrolide-resistant Mycoplasma pneumoniae pneumonia **(A)** nomogram; **(B)** calibration curve of the nomogram; **(C)** decision curve analysis; **(D)** joint ROC curve.

The predictive performance of individual variables for macrolide resistance, including the best cutoff values, sensitivity, specificity, and AUC, is summarized in Table 7. Among all indicators, PCT demonstrated the highest discriminatory ability with an AUC of 0.769. Based on the independent risk factors identified in the multivariate analysis, a nomogram was constructed to predict the individual probability of macrolide-resistant M. pneumoniae pneumonia (Figure 4). The calibration curve demonstrated good agreement between predicted and observed probabilities. The Hosmer-Lemeshow goodness-of-fit test yielded a non-significant *p*-value of 0.324, indicating no significant deviation from perfect calibration. Decision curve analysis further confirmed the clinical utility of the nomogram, showing a positive net benefit across a wide range of threshold probabilities. The combined predictive model incorporating these CT and serum markers demonstrated excellent discriminative ability, with a 10-fold cross-validated AUC of 0.869. The VIF for all 14 predictors ranged from 1.12 to 3.45 (all < 5), indicating no severe multicollinearity among the selected features.

**Table 7:** Predictive performance of CT and Serum markers for macrolide resistance.

Parameters	Best_threshold	Sensitivities (95% CI)	Specificities (95% CI)	AUC (95% CI)	Youden_index	F1_score
Fever Duration Pre-Admission (days)	5.695	0.593 (0.510–0.672)	0.731 (0.677–0.780)	0.685 (0.640–0.730)	0.324	0.560
Hospital Stay (days)	7.870	0.697 (0.617–0.769)	0.565 (0.506–0.622)	0.665 (0.619–0.711)	0.262	0.547
Shortness of Breath, *n* (%)	0.500	0.359 (0.282–0.443)	0.756 (0.703–0.803)	0.557 (0.509–0.605)	0.115	0.391
Oxygen Therapy Required, *n* (%)	0.500	0.255 (0.187–0.336)	0.862 (0.817–0.898)	0.559 (0.511–0.607)	0.117	0.335
CRP (mg/L)	45.305	0.379 (0.301–0.463)	0.866 (0.822–0.902)	0.633 (0.586–0.680)	0.245	0.462
D-dimer (mg/L FEU)	1.195	0.697 (0.617–0.769)	0.449 (0.391–0.508)	0.578 (0.530–0.626)	0.146	0.502
NLR	6.735	0.448 (0.366–0.533)	0.724 (0.669–0.773)	0.593 (0.546–0.640)	0.172	0.447
PCT (ng/mL)	0.485	0.648 (0.565–0.725)	0.806 (0.756–0.849)	0.769 (0.726–0.812)	0.454	0.639
IL-6 (pg/mL)	25.755	0.648 (0.565–0.725)	0.664 (0.607–0.718)	0.681 (0.635–0.727)	0.312	0.561
LAR	9.850	0.434 (0.353–0.519)	0.696 (0.640–0.747)	0.566 (0.518–0.614)	0.130	0.421
Lobar/Lobular Consolidation, *n* (%)	0.500	0.683 (0.602–0.755)	0.548 (0.489–0.606)	0.615 (0.568–0.662)	0.231	0.532
Bilateral Involvement, *n* (%)	0.500	0.662 (0.580–0.736)	0.484 (0.426–0.543)	0.573 (0.525–0.621)	0.146	0.496
Pleural Effusion, *n* (%)	0.500	0.324 (0.251–0.406)	0.855 (0.809–0.892)	0.590 (0.542–0.638)	0.179	0.403
CT Total Severity Score (TSS)	10.945	0.338 (0.264–0.421)	0.866 (0.822–0.902)	0.615 (0.568–0.662)	0.204	0.422
Nomogram (combined model)	0.498	0.786 (0.710–0.849)	0.803 (0.751–0.848)	0.869 (0.834–0.904)	0.589	0.773

## 4 Discussion

Macrolide resistance in Mycoplasma pneumoniae (M. pneumoniae) pneumonia poses a significant challenge in pediatric populations, necessitating more accurate methods for early identification and management. The present study aimed to develop a predictive nomogram by integrating CT imaging characteristics and serum biomarkers to assess the risk of macrolide resistance gene mutations in children with M. pneumoniae pneumonia, without relying on treatment response follow-up. This approach offers a comprehensive evaluation that goes beyond traditional clinical and laboratory assessments.

One of the key findings was that patients in the MRMP group experienced longer durations of fever prior to admission and extended hospital stays compared to those in the MSMP group. These prolonged periods suggest that MRMP may be associated with more severe inflammatory responses or delayed resolution of infection. This could be attributed to the persistence of bacterial pathogens despite macrolide treatment, leading to sustained immune activation and inflammation. The prolonged duration of fever might reflect the inability of macrolides to effectively inhibit the growth of resistant strains, thereby prolonging the course of illness and necessitating extended hospitalization (18–20).

The MRMP group exhibited a higher prevalence of shortness of breath and required oxygen therapy at admission more frequently than the MSMP group. Shortness of breath is indicative of respiratory distress, which can be exacerbated by the presence of lobar or lobular consolidation and pleural effusion, both of which were more common in the MRMP group. These findings suggest that macrolide-resistant infections may lead to more extensive lung involvement and compromised gas exchange (21, 22). The underlying mechanism could involve the failure of macrolides to suppress bacterial replication, resulting in increased lung tissue damage and subsequent respiratory complications. The need for oxygen therapy highlights the severity of respiratory compromise in MRMP cases, emphasizing the importance of early detection and targeted interventions (23, 24).

Serum levels of CRP, D-dimer, and the NLR were elevated in the MRMP group compared to the MSMP group. CRP is an acute-phase reactant that increases in response to inflammation, indicating a heightened systemic inflammatory response in MRMP patients. Elevated CRP levels could be due to the persistent bacterial burden and the resultant chronic inflammation, which may not be adequately controlled by macrolides. Similarly, increased D-dimer levels suggest enhanced fibrinolysis and coagulation activity, possibly reflecting the prothrombotic state induced by severe infection. The NLR, a marker of systemic inflammation, also showed higher values in the MRMP group, suggesting a more pronounced immune response. These elevated markers point to a more aggressive disease course in MRMP, driven by both bacterial persistence and dysregulated immune responses (25–27).

PCT and IL-6 levels were significantly higher in the MRMP group. PCT is a precursor of calcitonin that rises in response to bacterial infections, serving as a sensitive marker of sepsis and severe bacterial infections. The elevated PCT levels in MRMP indicate a more severe infection, potentially due to the inability of macrolides to control the bacterial load effectively. IL-6 is a pro-inflammatory cytokine involved in the acute phase response and plays a critical role in mediating inflammation. Higher IL-6 levels in MRMP suggest a more robust inflammatory response, contributing to the exacerbation of symptoms and lung pathology. The LAR was elevated in the MRMP group, indicating a shift towards a more pro-inflammatory state characterized by reduced lymphocyte function and increased CRP levels. These findings collectively highlight the profound impact of macrolide resistance on systemic inflammation and disease severity (28–30).

CT imaging revealed several notable differences between the MRMP and MSMP groups. Lobar/lobular consolidation, bilateral lung involvement, and pleural effusion were more frequent or severe in the MRMP group. These radiological features suggest more extensive lung involvement and greater tissue damage in MRMP. The mechanisms underlying these findings likely include the failure of macrolides to eradicate resistant bacteria, leading to persistent infection and ongoing lung injury. Bilateral lung involvement indicates widespread dissemination of the pathogen, while pleural effusion suggests the development of exudative processes secondary to the infection. The CT TSS was also higher in the MRMP group, reflecting the overall burden of lung pathology. These radiological observations underscore the need for alternative therapeutic strategies in managing MRMP to mitigate lung damage and improve outcomes (11, 31).

Several factors identified through univariate and multivariate analyses were confirmed as independent predictors of macrolide resistance. These included clinical signs such as shortness of breath and the need for oxygen therapy, elevated serum markers like CRP, D-dimer, NLR, PCT, IL-6, and LAR, and specific CT findings such as lobar/lobular consolidation, bilateral involvement, pleural effusion, and higher TSS. These predictors collectively provide a comprehensive risk profile for identifying MRMP patients. The integration of these factors into a predictive nomogram enhances the accuracy of risk stratification, facilitating timely and appropriate management decisions (32, 33).

The construction of the nomogram based on these independent risk factors offers a practical tool for predicting the likelihood of macrolide resistance in pediatric M. pneumoniae pneumonia. By combining CT imaging characteristics and serum biomarkers, the nomogram provides a more nuanced assessment of disease severity and resistance risk. This approach addresses the limitations of relying solely on clinical judgment or single biomarkers, offering a more holistic view of patient status. The predictive model demonstrated excellent discriminative ability, highlighting its potential utility in clinical practice (13, 34). The discriminative performance of our nomogram (AUC = 0.869) is comparable to that of a recently reported by Wang et al. (12) chest CT radiomics model for predicting macrolide resistance in pediatric MPP (AUC = 0.868) (12). However, our model incorporates both serum biomarkers and CT imaging features, providing a more comprehensive assessment that may offer practical advantages in clinical settings where radiomics analysis is not readily available.

In clinical practice, this nomogram is intended to be applied within 48 h of admission, utilizing routinely collected serum biomarkers and chest CT findings to estimate the probability of macrolide resistance. A predicted probability exceeding the optimal threshold (e.g., 0.5) may prompt clinicians to consider alternative antibiotics, such as tetracyclines or fluoroquinolones, pending further microbiological confirmation. This approach facilitates early, individualized therapeutic decisions, potentially reducing treatment failure and unnecessary macrolide exposure.

Notably, all 14 variables remained significant in the multivariable forward stepwise logistic regression model, indicating that each independently contributed to predicting macrolide resistance gene mutation status. This finding reflects the multifactorial nature of macrolide resistance in pediatric MPP, where clinical, laboratory, and imaging features collectively capture different dimensions of disease pathophysiology. No variable elimination was forced, as the stepwise selection criteria retained all candidates without evidence of multicollinearity.

Despite the promising results, this study has several limitations. The retrospective design limits the ability to establish causality between the identified risk factors and macrolide resistance. Prospective studies are needed to validate these findings and confirm their generalizability across different populations. While we accounted for various factors influencing macrolide resistance, other unmeasured variables may have influenced the results. Future research should aim to identify additional predictors and explore ways to optimize treatment protocols. The generalizability of our findings may be limited by the specific characteristics of our patient population. Studies involving diverse populations are necessary to validate the effectiveness of the combined monitoring strategies across different settings and patient groups. Although 10-fold cross-validation was performed to assess internal validity, the lack of an external validation cohort limits the generalizability of our nomogram. External validation in independent multicenter populations is needed to confirm its clinical utility. We plan to perform such validation in future studies. Additionally, this study only employed logistic regression to construct the nomogram; we did not compare its performance with other machine learning algorithms (e.g., random forest, support vector machine, or XGBoost). Future studies could explore these advanced modeling approaches to further improve predictive accuracy.

The integration of CT imaging characteristics and serum biomarkers offers a promising approach for predicting macrolide resistance in pediatric M. pneumoniae pneumonia. By leveraging these tools, clinicians can achieve a more accurate risk stratification and tailor interventions accordingly. Future research should focus on validating these findings and exploring the broader implications of this combined approach on patient outcomes and functional recovery. Enhanced understanding and application of these predictive models will contribute to improved management strategies and better patient care.

## 5 Conclusion

This study suggests that the integration of CT imaging characteristics and serum biomarkers may enhance the prediction of macrolide resistance gene mutation status in pediatric Mycoplasma pneumoniae pneumonia, providing a rapid alternative to genetic testing. Key factors including clinical symptoms, inflammatory markers, and specific radiological features collectively contribute to a more comprehensive risk assessment. The developed nomogram provides a potential tool for identifying patients at higher risk of macrolide resistance, facilitating timely and tailored therapeutic interventions. Further prospective studies are needed to validate these findings and to establish the optimal application of this combined approach in clinical practice. These preliminary observations indicate the potential for improved patient outcomes through enhanced risk stratification and personalized treatment strategies.

## Data Availability

The raw data supporting the conclusions of this article will be made available by the authors, without undue reservation.
